# Updates on the Diagnosis and Management of Glaucoma

**DOI:** 10.1016/j.mayocpiqo.2022.09.007

**Published:** 2022-11-16

**Authors:** Isabella V. Wagner, Michael W. Stewart, Syril K. Dorairaj

**Affiliations:** Department of Ophthalmology, Mayo Clinic School of Medicine, Jacksonville, FL

**Keywords:** ACA, anterior chamber angle, ACG, angle-closure glaucoma, AIT, ab-interno trabeculotomy, CAI, carbonic anhydrase inhibitor, CE, cataract extraction, GDD, glaucoma drainage device, IOP, intraocular pressure, KDB, Kahook Dual Blade, MIGS, minimally invasive glaucoma surgery, MMC, mitomycin C, OAG, open-angle glaucoma, OCT, optical coherence tomography, ONH, optic nerve head, PGA, prostaglandin analog, PGI, PAUL glaucoma implant, POAG, primary open-angle glaucoma, RNFL, retinal nerve fiber layer, SLT, selective laser trabeculoplasty, TM, trabecular meshwork

## Abstract

Glaucoma is the leading cause of blindness throughout the world (after cataracts); therefore, general physicians should be familiar with the diagnosis and management of affected patients. Glaucomas are usually categorized by the anatomy of the anterior chamber angle (open vs narrow/closed), rapidity of onset (acute vs chronic), and major etiology (primary vs secondary). Most glaucomas are primary (ie, without a contributing comorbidity); however, several coexisting ophthalmic conditions may serve as the underlying etiologies of secondary glaucomas. Chronic glaucoma occurs most commonly; thus, regular eye examinations should be performed in at-risk patients to prevent the insidious loss of vision that can develop before diagnosis. Glaucoma damages the optic nerve and retinal nerve fiber layer, leading to peripheral and central visual field defects. Elevated intraocular pressure (IOP), a crucial determinant of disease progression, remains the only modifiable risk factor; thus, all current treatments (medications, lasers, and operations) aim to reduce the IOP. Pharmacotherapy is the usual first-line therapy, but noncompliance, undesirable adverse effects, and cost limit effectiveness. Laser and surgical treatments may lower IOP significantly over long periods and may be more cost effective than pharmacotherapy, but they are plagued by greater procedural risks and frequent treatment failures. Traditional incisional procedures have recently been replaced by several novel, minimally invasive glaucoma surgeries with improved safety profiles and only minimal decreases in efficacy. Minimally invasive glaucoma surgeries have dramatically transformed the surgical management of glaucoma; nevertheless, large, randomized trials are required to assess their long-term efficacy.

**Article Highlights**

• Glaucoma, a leading cause of blindness throughout the world, presents with an open or closed anterior chamber angle, structural damage to the optic nerve (seen in all stages), and visual field defects (seen in later stages). Glaucoma may be asymptomatic until the late stages, thereby emphasizing the need for general physicians to understand important diagnostic criteria and management options.
• The progression of glaucoma is mitigated by lowering the intraocular pressure, which is done with topical medications, laser procedures, or incisional operations.
• Minimally invasive glaucoma surgery, with a favorable safety profile and efficacy that rivals traditional incisional procedures, has transformed glaucoma care.

Glaucoma can be defined as a progressive optic neuropathy that induces optic disc cupping and retinal ganglion cell apoptosis.^1^ As the world’s leading cause of irreversible blindness, the disease currently affects 3.5% of individuals aged between 40 and 80 years. The incidence of glaucoma is increasing, together with life expectancies, in resource-limited countries, and nearly 112 million people are expected to be affected by 2040.^1^^,^^2^ Early detection can slow disease progression, but because visual field loss may be asymptomatic until the late stages, a timely diagnosis is frequently delayed.^3^ Common risk factors for glaucoma include advancing age, a positive family history, race (non-Caucasian), and elevated intraocular pressure (IOP).^4^^,^^5^ Once diagnosed with glaucoma, most patients require lifelong care.

Aqueous humor is produced by the ciliary body, and after percolating through the posterior chamber, around the lens, and through the pupil, it exits the eye through the semiporous trabecular meshwork (TM) in the iridocorneal angle of the anterior chamber. Aqueous humor then flows into the circumferential vascular collection duct (Schlemm canal) and leaves the eye through the distal collector channels that drain into the episcleral venous system.^6^, ^7^, ^8^ A detailed anatomical view of the anterior eye segment and the aqueous outflow pathway is displayed in Figure 1. The pathogenesis of glaucoma includes inadequate drainage of aqueous humor because of increased resistance through the meshwork^7^ or occlusion of the angle,^9^ both of which elevate the IOP. Elevated IOP contributes to an irreversible, progressive ocular neuropathy characterized by retinal ganglion cell apoptosis.^1^ Patients with elevated IOP without other signs of glaucoma are said to have ocular hypertension, and those with optic disc enlargement but normal IOP and no other signs of glaucoma are classified as glaucoma suspects.

**Figure 1:**
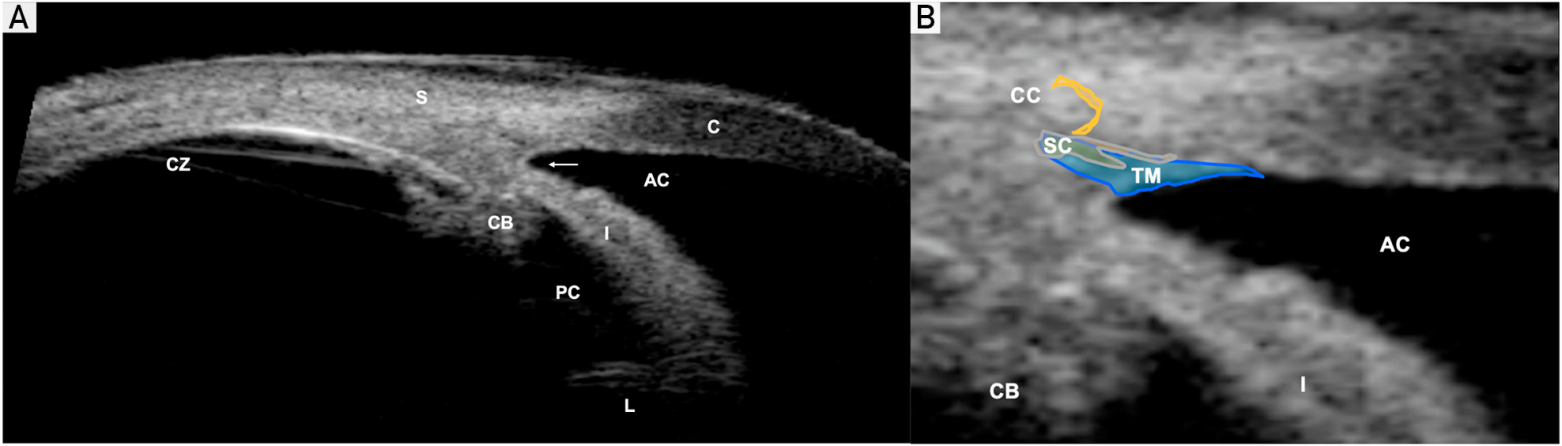
Ultrasound biomicroscopy (UBM) of the anterior eye segment. A, UBM shows the ciliary zonules (CZ), ciliary body (CB), sclera (S), cornea (C), anterior chamber (AC), posterior chamber (PC), and lens (L). The anterior chamber angle (ACA) is indicated by the arrow. B, Magnified UBM of the ACA shows the trabecular meshwork (TM), Schlemm canal (SC), and collector channels (CCs).

The risk factors and pathogenesis that underly glaucoma have been well described in the literature; however, the biological basis of the disease remains incompletely understood. The biomechanical and vascular theories of glaucoma propose that elevated IOP compromises axonal integrity at the optic nerve head (ONH), which leads to ganglion cell apoptosis.^5^ The biomechanical theory posits that abnormally narrow scleral fenestrations at the ONH limit axoplasmic flow,^5^^,^^7^^,^^10^ whereas the vascular theory states that decreased perfusion pressure leads to hypoxia and ischemic damage of the ONH.^5^^,^^7^^,^^11^ Both theories include IOP as a risk factor; however, one-third of patients with glaucoma have normal IOPs (normal tension glaucoma).^5^ Glaucoma has been associated with Alzheimer disease^12^ and a loss of cognitive function,^13^ which suggests that neurodegeneration may contribute to the pathogenesis.^5^ However, despite the different pathogenetic theories, elevated IOP consistently contributes to disease progression and remains the only treatable risk factor.^5^^,^^7^

The goal of glaucoma treatment is to lower IOP with medications, laser procedures, and/or operation. First-line therapy is usually pharmacotherapy, with laser and surgical procedures added for further IOP reduction in eyes with inadequate initial responses. Incisional operations consist of filtration procedures (eg, trabeculectomy) or tube shunt implantation, both of which reroute aqueous humor flow past the damaged angle into the subconjunctival space forming a filtration bleb.^14^

Traditional incisional operations lower the IOP effectively; however, complication rates, including scar tissue proliferation, endophthalmitis, and conjunctival hemorrhage, are high. The IOP-lowering effect often decreases over time, which results in high 5-year reoperation rates (trabeculectomy, 15.1%; tube shunt implantation, 14.0%; EX-PRESS shunt, 18.3%).^15^, ^16^, ^17^ These high reoperation rates speak to the need for procedures that increase conventional aqueous outflow while protecting the conjunctiva from surgical manipulation. This has led to the development of several conjunctival sparing, minimally invasive glaucoma surgeries (MIGSs) for the treatment of primary open-angle glaucoma (POAG). Minimally invasive glaucoma surgeries do not reduce IOP as well as traditional filtering procedures, but they have excellent safety profiles.^18^

We believe that because of the expanding treatment options and increasing worldwide prevalence of glaucoma, an updated commentary on glaucoma and its treatment options is important for medical physicians. In this article, we provide a comprehensive updated review of the diagnosis and management of adult glaucoma through 2022.

## Methods

A broad literature search with no time frame was carried out in PubMed with the following key words: “glaucoma prevalence,” “glaucoma risk factors,” “glaucoma diagnosis,” “glaucoma management,” “open-angle glaucoma,” angle-closure glaucoma,” “secondary glaucoma,” “tonometry,” “glaucoma medication,” “conventional aqueous outflow,” “unconventional aqueous outflow,” “glaucoma laser procedures,” “trabeculectomy,” “glaucoma tube shunt surgery,” and “minimally invasive glaucoma surgery.” Identified articles and their references were scrutinized, and those relevant to the subject matter were selected.

## Diagnosis of Glaucoma

### Types of Glaucoma

Glaucoma may be broadly categorized as open-angle glaucoma (OAG) and angle-closure glaucoma (ACG). Primary OAG and primary ACG are seen most frequently; however, several ocular conditions cause secondary glaucomas (Table 1).

**Table 1:** Common Glaucoma Types are Listed According to Whether the Anterior Chamber Angle is Open or Closed^a^

Glaucoma type	Clinical features
Open-angle glaucoma	Normal iridocorneal angle; no iris occlusion
Primary open angle (includes normal tension glaucoma)	• ONH degeneration and decrease of aqueous outflow with no apparent etiology
Pigmentary	• Widespread deposition of pigment within the iris and corneal endothelium • Homogenous pigmentation of TM • Transillumination defects of iris
Exfoliative	• Deposition of exfoliative, dandruff-like material onto the anterior segment structures (eg, zonules, pupillary margin, TM, anterior lens surface) • Accelerated visual deterioration
Uveitic^b^	• Anterior chamber inflammation; excessive elevation of IOP • Preperimetric, mild optic disk changes
Traumatic	• Premature cataract after blunt-force trauma • Angle recession • Hyphema
Induced by steroids	• IOP spike after the use of topical/systemic steroids • Increased production of extracellular matrix material (elastin, type IV collagen, and glycosaminoglycans) • Frequently asymptomatic
Induced by antineoplastic drugs	• IOP spike after the use of taxane agents (docetaxel, paclitaxel)
Induced by increased episcleral venous pressure	• Dilated episcleral veins • Resistance to antiglaucoma medications
Angle-closure glaucoma	Closed iridocorneal angle; iris occlusion
Primary angle closure	• Appositional angle closure (pupillary block) or observed contact between TM and iris (plateau iris)
Neovascular	• Neovascularization within the anterior segment and over the iridocorneal angle • Retinal ischemia • Poor visual prognosis
Phacomorphic	• Presence of a thick, mature cataract
Induced by iridocorneal endothelial syndrome	• Secondary corneal edema • Iris stroma irregularities • Peripheral anterior synechiae • Resistance to antiglaucoma medications
Induced by iris tumor/ciliary body tumor/Soemmering ring	• Synechial angle narrowing because of mass enlargement • Opacification of the posterior capsule • Pupillary block
Induced by medications	• Pupillary block–induced angle closure after the use of adrenergic agonists and anticholinergic agents • Plateau iris–induced angle closure after the use of cholinergic and sulfonamide agents

a IOP, intraocular pressure; ONH, optic nerve head; TM, trabecular meshwork.

b Can be associated with an open or closed iridocorneal angle.

Most eyes with glaucoma have diminished conventional aqueous outflow despite a normal gonioscopic appearance of the iridocorneal angle. These OAGs are slowly progressive optic neuropathies in which ONH cupping gradual increases and peripheral visual field loss develops.^15^^,^^19^ The most common type of glaucoma—the POAG—affects 74% of patients with glaucoma.^20^ Outflow resistance may be modulated by hydrodynamic pore-substrate interactions within the inner wall of the Schlemm canal, and patients with POAG have been found to have reduced pore density.^21^

Several types of secondary OAG occur much less frequently than POAG. Pigmentary glaucoma occurs when friction between the lens zonules and iris pigment epithelium releases pigment granules that lodge in the TM and increase outflow resistance.^22^^,^^23^ Exfoliative glaucoma, the most common form of secondary OAG, occurs when microscopic clumps of protein fibers are synthesized within the eye and clog the TM.^24^ Exfoliation material has also been found in the heart, kidney, liver, and lungs.^24^^,^^25^ Other forms of secondary OAG include uveitic and traumatic glaucomas,^26^, ^27^, ^28^ use of ocular or systemic corticosteroids,^29^ and antineoplastic drugs.^30^ Increased episcleral venous pressure due to conditions such as carotid-cavernous sinus fistulas may cause OAG.^31^

Angle-closure glaucomas are rapidly progressive ocular neuropathies characterized by the occlusion of at least 270° of the iridocorneal angle.^3^ Angle-closure glaucomas are only one-third as common as OAGs; however, they are responsible for approximately 50% of all glaucoma-induced blindness. Primary ACG, which arises from pupillary block (appositional closure of the iridocorneal angle that results from an increasing pressure differential between the anterior and posterior chambers of the eye^32^) or plateau iris (an anteriorly positioned ciliary body that causes contact between the iris and TM with resultant angle crowding^33^), has a global prevalence of 0.6%.^3^^,^^34^^,^^35^ Primary ACG occurs most frequently in women, Asians, people with hypermetropic (short) eyes and people with shallow anterior chambers.^34^ Affected patients require urgent treatment (usually laser iridotomy) to reverse obstruction of the angle.^34^

Several secondary types of ACG are seen. Neovascular glaucoma, new blood vessels that occlude the angle, may develop from central retinal vein occlusion or diabetic retinopathy and generally carries a poor visual prognosis.^1^^,^^36^ Phacomorphic glaucoma involves angle-closure because of lens intumescence (advanced cataract), and cataract removal typically leads to good visual recovery.^37^ Angle-closure may be caused by corneal endothelium abnormalities (eg, iridocorneal endothelium syndromes)^38^ or large iris or ciliary body masses.^39^ Several medications, including anticholinergics, may precipitate ACG in eyes with preexisting narrow angles.^1^^,^^40^

Differentiating between OAG and ACG is usually done via gonioscopic examination with slit lamp viewing.^41^ Gonioscopy has long been the gold standard for visualizing the anterior chamber angle (ACA); however, challenges, including lens-eye contact, lack of objective measurements, a steep learning curve, and inconsistent interpretations between physicians, exist.^41^^,^^42^ Advanced ACA imaging techniques including swept-source optical coherence tomography (OCT), goniophotography systems, and deep learning algorithms have been developed to overcome the limitations of gonioscopy.^43^

### Examination

Approximately 50% of individuals in the resource-limited countries are unaware that they have glaucoma, underscoring the importance of patient awareness education in diagnosis and management.^3^^,^^44^^,^^45^ The diagnosis of glaucoma involves risk assessment, measurement of visual acuity, IOP, and corneal thickness, OCT imaging of the retinal nerve fiber layer (RNFL) and ONH, and visual field testing. Because most patients with glaucoma are asymptomatic for years, it is recommended that those with risk factors (advanced age, family history, non-White race, high IOP, and steroid use) be referred to an eye care provider for a glaucoma assessment.^3^, ^4^, ^5^

Intraocular pressure needs to be monitored regularly in patients at a high risk of developing glaucoma. It is commonly measured using rebound tonometry (iCare ic100; iCare) or the “gold standard” Goldmann applanation tonometry. The iCare tonometer measures IOP-dependent rebound velocity after brief corneal contact, whereas Goldmann applanation tonometry measures the force required to flatten a 3.06-mm diameter segment of the cornea.^46^ Agreement in measurements is good between the 2 devices; however, the reliability of the iCare decreases at higher IOPs and with thicker central corneas.^47^, ^48^, ^49^ Normal IOP ranges from 11 to 21 mm Hg^50^; however, IOP should be evaluated with consideration of optic nerve defects and/or high central cornea values.^51^ Up to 50% of glaucomatous eyes have normal IOP measurements,^3^^,^^52^ which emphasizes the importance of performing additional diagnostic imaging when indicated.

**Figure 2:**
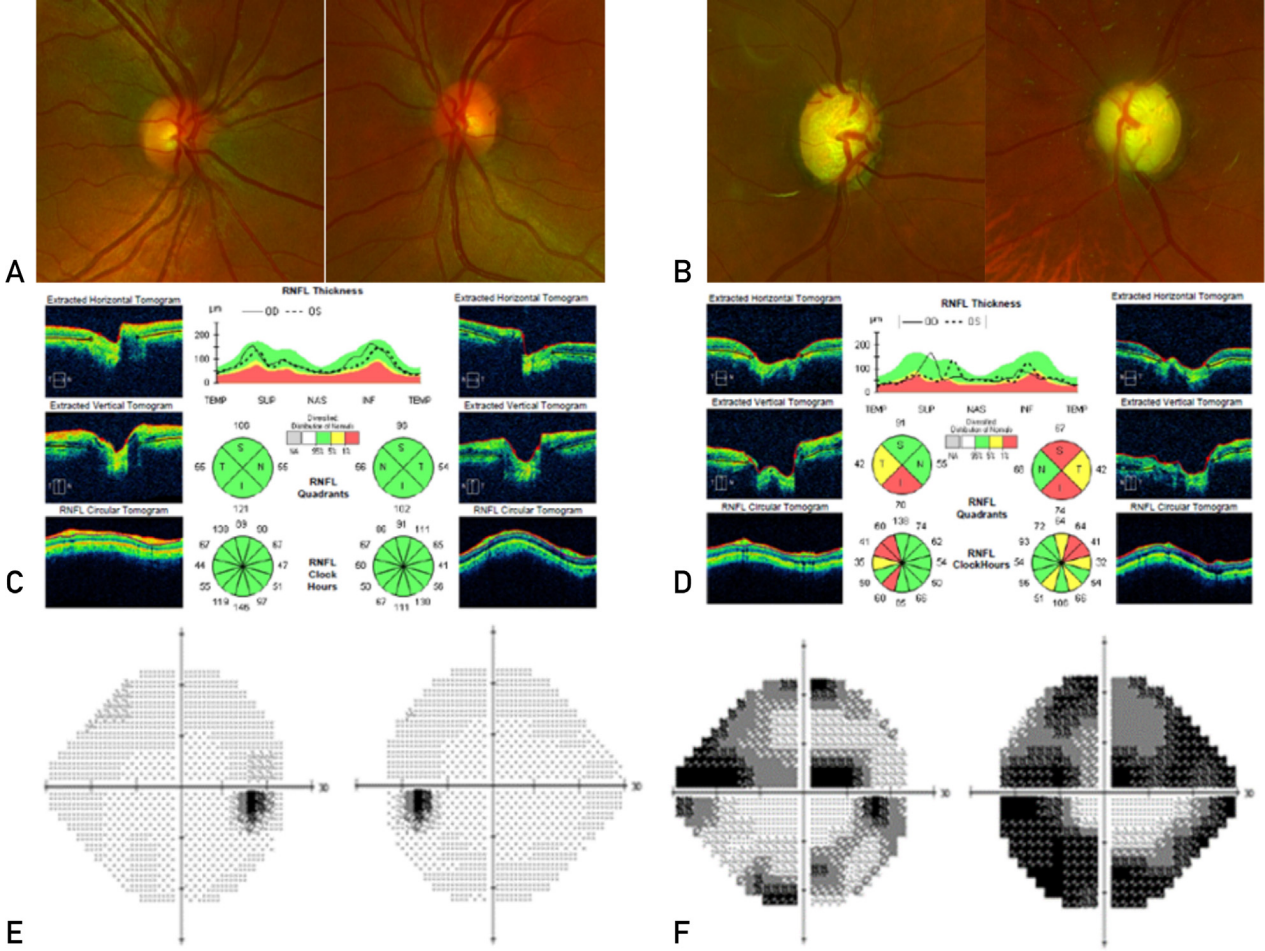
Comparison of optic nerve head (ONH), retinal nerve fiber layer (RNFL), and visual fields in normal and glaucomatous eyes. A, Normal ONH with round, elevated ONH and a small central cup. B, Glaucomatous ONH with excavation and thinning of neuroretinal rim. C, Optical coherence tomography (OCT) examination shows normal RNFL thickness. D, OCT examination shows RNFL thinning in glaucomatous eyes. E, A full field in both eyes is shown. F, Abnormal visual field results in glaucomatous eyes are shown. The right eye field shows a superior altitudinal defect, moderate inferior arcuate defects, and a nasal step. The left eye field shows a superior paracentral defect with nasal step that splits fixation, an early inferior arcuate scotoma, and nasal step.

Making the diagnosis of glaucoma, particularly at an early stage, can be difficult because there is no uniform standard for diagnosis.^3^ Structural changes of early glaucoma can be seen with OCT imaging of the optic nerve and macula, and functional changes in advanced glaucoma can be detected with visual field testing. Normal appearances of the ONH, RNFL, and visual field are shown in Figure 2A, C, and E, respectively. All glaucomas are defined by ONH degeneration with disc excavation (Figure 2B) and RNFL thinning (Figure 2D).^53^ Optic nerve head damage is characterized by thinning of the neuroretinal rim, usually in the superior and inferior quadrants, although the remainder of the ONH may remain pink with a normal neuroretinal rim.^3^^,^^53^ Glaucomatous damage leads to retinal ganglion cell apoptosis, which can be seen as thinning between the internal limiting membrane and ganglion cell layer on OCT.^53^ As glaucoma progresses, ONH and RNFL abnormalities cause visual field defects (Figure 2F). Visual field defects are often not observed in the early stages of glaucoma because peripheral vision and Snellen visual acuity are preserved until RNFL damage reaches an advanced stage.^51^

A general correlation between OCT imaging and visual field examination can be observed; however, there is no widely accepted method for comparing the two,^54^ and diagnosing glaucoma is ultimately up to the discretion of the physician. Once glaucoma has been diagnosed, its severity must be categorized—typically as mild, moderate, or severe. Because all glaucoma types present with structural damage, most classification systems grade severity on the basis of functional visual field abnormalities. Most recently (2015), the *International Classification of Diseases, Tenth Revision,* released a grading system that associates mild glaucoma with a general absence of visual field defects, moderate glaucoma with visual field abnormalities in 1 hemifield (but outside 5° of fixation), and severe glaucoma with abnormalities in both hemifields and visual field loss within 5° of fixation.^55^

## Management of Glaucoma

### Medical Therapy

Guidelines from the American Academy of Ophthalmology Preferred Practice Pattern (2020) state that an initial IOP reduction of 20%-30% is a suitable goal to slow disease progression, even in eyes with normal tension glaucoma.^56^ The IOP must be carefully monitored during each follow-up visit, and the IOP control goal should be lowered further if progression continues.^56^

Intraocular pressure–lowering medications have been the first-line therapy for most patients with glaucoma for several decades (Table 2). Pharmacotherapy for glaucoma has evolved significantly over the past several decades with the introduction of topical carbonic anhydrase inhibitors (CAIs), beta blockers, prostaglandin analogs, and alpha agonists.^57^ These medications have greater effectiveness and more favorable safety profiles than the older topical (pilocarpine) and systemic (oral CAIs) treatments.^57^ In accordance with the generally accepted pharmacotherapy principles, the desired IOP range should be achieved with the fewest medications and least adverse effects.^3^ Because of their tendency to induce glaucoma, ocular and systemic corticosteroids should be administered with caution in at-risk patients.^29^

**Table 2:** US Food and Drug Administration–Approved Medications Used for the Treatment of Glaucoma

Class	Medications^a^	Adverse effects	Contraindications
Prostaglandin analogs	• Bimatoprost • Latanoprost • Tafluprost • Travoprost • Unoprostene • Latanoprostene Bunod	• Eyelash growth • Iris darkening • Keratitis • Conjunctival keratitis • Uveitis	• Hypersensitivity to ingredients
Cholinergic agonists	• Pilocarpine • Carbachol	• Myopia • Angle closure • Cataract • Retinal detachment	• Miosis • Bradycardia • Retinal detachment • Asthma • Inflammatory eye disease
Carbonic anhydrase inhibitors	First generation (systemic): • Acetazolamide • Methazolamide • Dichlorphenamide Second generation (topical): • Brinzolamide • Dorzolamide	First generation (systemic): • Renal calculi • Stevens-Johnson syndrome • Serum electrolyte imbalance Second generation (topical): • Corneal edema • Metallic taste	• Allergy to sulfa-containing medications (both) • Adrenal insufficiency, metabolic acidosis (systemic inhibitors only) • Sickle cell disease (topical inhibitors only)
Beta adrenergic antagonists	Nonselective: • Carteolol • Levobunolol • Metipranolol • Timolol β1-selective: • Betaxolol	• Congestive heart failure • Exercise intolerance • Hypotension • Bronchospasm • Bradycardia	• Cardiovascular disease • Asthma • Diabetes mellitus • Chronic obstructive pulmonary disease
Αlpha adrenergic agonists	• Apraclonidine • Brimonidine	• Hypotension • Fatigue • Allergic conjunctivitis	• Monoamine oxidase inhibitor therapy
Rho kinase inhibitors	• Netarsudil	• Keratitis • Conjunctival hemorrhage • Corneal verticillata	• None
Hyperosmotic agents	• Glycerol • Mannitol • Isosorbide	• Congestive heart failure • Renal failure • Nausea • Vomiting • Headache	• Cardiovascular disease • Renal failure

a Common antiglaucoma medications decrease the intraocular pressure by decreasing aqueous humor production or increasing outflow.

Prostaglandin analogs (PGAs) are the most commonly used medications for the treatment of OAG and ocular hypertension. Prostaglandin analogs compensate for decreased TM outflow by increasing outflow through the uveoscleral pathway,^58^ where aqueous humor moves through the ciliary muscle into the supraciliary and suprachoroidal spaces.^59^ Prostaglandin analogs are administered once daily, are well tolerated, and have limited systemic adverse effects.^3^^,^^58^ The main ocular adverse effects are eyelash growth, iris pigmentation, and uveitis.^56^ Because most PGAs do not target the primary outflow pathway (TM), concerns have been raised about their long-term efficacy.^57^ The recently approved latanoprostene bunod 0.024% may target the TM rather than the uveoscleral pathway,^57^^,^^60^ and compared with timolol 0.5% over 3 months of follow-up, it has superior IOP-lowering efficacy and a comparable safety profile.^57^^,^^61^^,^^62^ Prostaglandin analogs are a significant improvement over cholinergic agonists (such as pilocarpine), which induce miosis and increase conventional outflow by decreasing outflow resistance.^63^ Pilocarpine, a mainstay of glaucoma treatment in the 1970s and 1980s, needed to be administered 4 times per day, a difficult regimen to maintain, which contributed to its being supplanted by beta blockers and PGAs.^3^

Both CAIs and beta blockers lower the IOP by targeting the aqueous humor production in the ciliary body. After topical administration, CAIs penetrate the cornea and reach the ciliary body epithelium, where they reduce the production of bicarbonate ions.^64^ The CAIs (dorzolamide 2% and brinzolamide 1%) are administered 2 or 3 times daily,^64^ but they are generally less effective than PGAs and beta blockers, which limits their use as first-line therapy. Systemic CAIs (methazolamide and acetazolamide) are highly effective, which makes them useful in the treatment of ACG; however, their use is limited by their high incidence of adverse effects that cause 50% of patients to become intolerant after 1 month.

Beta adrenergic antagonists (beta blockers) block the sympathetic nerve endings in the ciliary body epithelium, which decreases the production of aqueous.^65^ Beta blockers may be nonselective or cardioselective (β1 selective), the latter of which is well tolerated in patients with asthma and chronic obstructive pulmonary disease.^65^ The advantages of beta blockers include their relatively low cost and once-daily administration.^3^^,^^5^ Topically administered beta blockers enter the venous circulation but escape the first-pass metabolism in the liver, which predisposes the patient to pulmonary (bronchial constriction) and cardiac (arrythmias) disturbances.^5^^,^^66^ Systemic absorption can be lessened by eyelid closure or gentle punctal occlusion for 2 minutes after topical administration.^3^

Topical alpha-adrenergic agonists (brimonidine and iopidine) reduce the IOP by decreasing the aqueous humor production and increasing the outflow.^3^ They are administered 2 or 3 times daily and are usually used as second-line agents in combination with other drugs. A retrospective study found that combination treatment (CAI+PGA) was more prevalent in everyday practice than alpha-2 agonists + PGA, suggesting that the administration of alpha-2 agonists may be accompanied by more adverse effects.^67^

Rho kinase inhibitors are a recently introduced medication class that uses a combined mechanism of increasing the conventional outflow and decreasing the episcleral venous pressure.^68^ Netarsudil 0.02%, a rho kinase inhibitor approved by the US Food and Drug Administration in 2017, has IOP-lowering efficacy comparable with that of timolol 0.5%, but with more frequent adverse effects.^59^^,^^69^^,^^70^

Pharmacotherapy is an effective short-term treatment strategy; however, limitations to long-term use include cost, adverse effects, and failure to reach the target IOP. Nonadherence to the administration schedule is another significant issue because fewer than half of the patients with glaucoma regularly use antiglaucoma medications as prescribed after 1 year.^5^^,^^71^

### Laser Therapy

When pharmacotherapy fails to achieve the target IOP and prevent vision loss, laser and surgical procedures are indicated. Laser procedures effectively lower the IOP and minimize the long-term costs that are associated with the long-term use of multiple pressure-lowering medications.^5^ A variety of laser procedures can be performed in glaucomatous eyes, with the procedure of choice depending on the etiology of the disease (Table 3).

**Table 3:** Laser Procedures for the Treatment of Glaucoma

Laser procedure	Preferred use	Pros	Cons
Laser trabeculoplasty • Argon laser trabeculoplasty • Selective laser trabeculoplasty • MicroPulse laser trabeculoplasty • Titanium-sapphire laser trabeculoplasty • Pattern scanning trabeculoplasty	• Open-angle glaucoma	• Performed in-office • Minimally invasive • Newer methods protect the TM tissue	• Decrease in efficacy over time • May cause transient IOP spikes and anterior uveitis
Excimer laser trabeculostomy	• Open-angle glaucoma	• Minimally invasive • Minimizes tissue fibrosis	• Performed in the operating room • Requires incision
Laser peripheral iridotomy	• Angle-closure glaucoma (pupillary block)	• Performed in-office • Highly effective in the treatment of pupillary block–induced angle closure	• Not sufficient to relieve the angle closure caused by multiple mechanisms • May promote cataract progression
Laser peripheral iridoplasty	• Angle-closure glaucoma (plateau iris)	• Performed in-office • Can relieve appositional angle closure after an LPI • Effective in the treatment of angle closure caused by multiple mechanisms	• May cause atrophic iris scarring and loss of visual acuity • May develop Urrets-Zavalia syndrome
Cyclodestructive procedures • Endoscopic cyclophotocoagulation • Continuous-wave diode transscleral laser • MicroPulse diode transscleral laser therapy	• Glaucoma refractory to surgical treatment • Secondary glaucoma	• High IOP-reducing efficacy from mechanism targeting ciliary body	• Associated with a series of complications (hyphema, macular edema, mydriasis, decrease in visual acuity, keratitis, etc) • May require multiple treatments • Performed in the operating room

IOP, intraocular pressure; LPI, laser peripheral iridotomy; TM, trabecular meshwork.

Laser trabeculoplasty and ab-interno excimer trabeculostomy (Glautec AG) are both indicated for OAG that is refractory to pharmacotherapy. Laser trabeculoplasty—multiple spots of thermal laser applied directly to the TM—induces favorable structural changes that increase the aqueous humor outflow.^72^ Argon laser trabeculoplasty, developed in 1979, uses a with a blue-green continuous-wave laser (488 and 514 nm) to disrupt the TM, whereas selective laser trabeculoplasty (SLT), developed in 1995, uses low energy, brief duration, large spots from a green, frequency-doubled laser to target melanin-containing cells and spare the TM tissue.^73^ Selective laser trabeculoplasty has largely supplanted argon laser trabeculoplasty because of its favorable safety profile, comparable IOP-lowering efficacy, and ability for repeated treatment applications.^74^ More recently introduced laser trabeculoplasty procedures include titanium-sapphire laser trabeculoplasty and pattern scanning trabeculoplasty. Limited short-term data suggest that both the procedures have efficacy and safety profiles similar to that of SLT.^74^ Laser trabeculoplasty procedures are generally preferred over operations because they are less invasive and possess better safety profiles.^3^^,^^74^ Ab-interno excimer trabeculostomy is a MIGS similar to laser trabeculoplasty that uses a 308-nm XeCl excimer laser to create microperforations in the TM and inner wall of the Schlemm canal.^75^ Excimer trabeculostomy has a comparable safety profile and IOP-lowering efficacy similar to SLT over 2 years.^75^

Patients with ACG require different laser procedures from those with OAG. A laser peripheral iridotomy creates a hole in the peripheral iris and is often performed to eliminate pupillary block,^76^ whereas a laser peripheral iridoplasty uses low-power laser burns to relieve appositional angle closure (by shrinking the peripheral iris) in cases where laser peripheral iridotomy is ineffective.^77^ When combined, both treatments have been shown to be safe and effective in lowering the IOP in eyes with acute primary ACG refractory to pharmacotherapy.^78^ For eyes refractory to all other medical, surgical, and laser therapies, a series of cyclodestructive procedures that damage the ciliary body epithelium and decrease the IOP by reducing the aqueous humor secretion may be the final treatment option.^79^ These procedures consist of endoscopic cytophotocoagulation (Endo Optiks), continuous-wave diode laser (IRIDEX Corp), or the newest alternative, MicroPulse transscleral laser therapy (IRIDEX Corp), which selectively targets the pigmented tissue of the ciliary body epithelium.^79^ Cyclodestructive procedures are also useful for the secondary forms of glaucoma, such as uveitic, traumatic, or neovascular glaucoma; however, these procedures come have considerable risks and are particularly difficult to titrate.^79^

### Surgical Treatment

Operations are usually performed when medical and laser treatments have failed to achieve adequate IOP reduction. Surgical options consist of the traditional, bleb-based IOP-lowering operations (trabeculectomy and tube shunt implantation) and the newer, conjunctiva-sparing MIGSs (Table 4). Bleb-based operations can effectively lower IOP; however, they may develop bleb-related complications and may have high reoperation rates. As a result, the current role of traditional procedures in the era of evolving MIGSs is unclear. Surgeons’ perspectives are changing^80^; a recent practice preferences survey from the American Glaucoma Society (2017) found that trabeculectomy has fallen out of favor, with tube shunt implantation reported as the preferred incisional surgical treatment in 7 of 8 surgical centers.^81^ When prospective MIGS trials are completed, the pendulum may swing in favor of MIGSs.^80^

**Table 4:** Surgical Procedures for the Treatment of Glaucoma^a^

Procedure^b^	Type	Pros	Cons
Trabeculectomy	• Incisional operation • Antimetabolite-associated	• Excellent IOP control • Can adjust the rate of fluid flow	• Bleb-related complications
Ex-PRESS mini shunt operation	• Incisional operation	• Favorable safety profile to trabeculectomy • Minimal IOP fluctuations	• Bleb-related complications • High incidence of erosion, displacement, and hypotony
Valved drainage implants • Ahmed FP7 valve • Ahmed FP8 valve • Pars plana Ahmed	• Incisional operation	• Immediate IOP reduction • Valve reduces hypotony-associated complications during early postoperative period	• Bleb-related complications • Malfunctioning of the valve may result in hypotony
Nonvalved drainage implants • Molteno glaucoma drainage device • Baerveldt glaucoma implant • Ahmed ClearPath drainage device • PAUL glaucoma implant	• Incisional operation	• Greater surface area promotes sustained reduction of IOP	• Bleb-related complications • Delayed encapsulation and high incidence of hypotony in older Molteno and Baervedlt models
Trabecular bypass • iStent • iStent inject • iStent inject W • Hydrus Microstent	• MIGSs targeting the trabecular outflow pathway	• Low risk of hypotony • Favorable safety profile • Effective for mild and moderate glaucoma	• Does not achieve IOP reduction comparable to trabeculectomy • Not suitable for severe glaucoma • High risk of fibrosis
Canaloplasty • Ab-externo canaloplasty without tensioning suture • Ab-externo canaloplasty with tensioning suture • ABiC	• MIGSs targeting the trabecular outflow pathway	• Low complications rates • ABiC: safer and easier than ab-externo approach	• Generally not suitable for severe glaucoma
Ab-interno trabeculotomy; goniotomy • Trabectome • Goniotome • Gonioscopy assisted transluminal trabeculotomy • iAccess (Glaukos) • Kahook Dual Blade goniotomy • Kahook Dual Blade Glide	• MIGSs targeting the trabecular outflow pathway	• Goniotomy: clean excision of TM limits fibrosis and closure	
Trabeculotomy/viscodilation • OMNI Surgical System	• MIGSs targeting the trabecular outflow pathway	• Targets all 3 points of outflow resistance (TM, Schlemm canal, collector channels)	
Goniotomy/viscodilation • STREAMLINE Surgical System	• MIGSs targeting the trabecular outflow pathway	• Ease of use • Combined TM excision and delivery of viscoelastic promotes high IOP reduction	• No long-term efficacy • Potential risk of fibrosis
Ab-interno subconjunctival implant • XEN45 gel stent • PRESERFLO microshunt	• MIGSs targeting the subconjunctival space	• Greater IOP-lowering efficacy than angle-based MIGS • Suitable for severe glaucoma	• Bleb-related complications • Subconjunctival fibrosis
Ab-interno suprachoroidal implant • CyPass MicroStent (withdrawn) • iStent SUPRA^c^ • STARflo glaucoma implant^c^ • MINIject^c^ • SOLX gold shunt (SOLX, Inc)^c^	• MIGSs targeting the suprachoroidal space	• Greater IOP-lowering efficacy than angle-based MIGS	• High risk of transient IOP spikes and fibrosis

a IOP, intraocular pressure; MIGS, minimally invasive glaucoma surgery; TM, trabecular meshwork.

b Procedures have been divided into traditional filtration operations (creation of a scleral flap and filtration bleb) and newly emerging microinvasive glaucoma operations.

c Still in development.

### Trabecular Outflow Resistance

The juxtacanalicular tissue within the TM is the primary source of outflow resistance in eyes with POAG, with the inner wall of the Schlemm canal serving as an additional line of resistance.^82^, ^83^, ^84^ To improve the aqueous outflow and lower the IOP, surgeons bypass the TM by directing the aqueous flow directly into the Schlemm canal or by rerouting the fluid from the anterior chamber into the subconjunctival space.

### Traditional Incisional Operations

Trabeculectomy—the “gold standard” surgical glaucoma procedure for several decades—is the creation of a partial thickness scleral flap with excision of a segment of TM to create an alternate drainage route from the anterior chamber to the subconjunctival space.^85^^,^^86^ Trabeculectomy can produce outstanding IOP control, particularly in eyes where an IOP near the low teens is targeted to slow glaucoma progression.^87^^,^^88^ Trabeculectomy may be performed together with cataract extraction (CE) and/or administration of mitomycin C (MMC) on the surface of the sclera to prevent postoperative conjunctival fibrosis.^89^ Trab-MMC alone, trab-MMC+CE, and trab-MMC in pseudophakic eyes were found to produce comparable IOP reductions and success rates after 5 years^90^; however, other studies have found lower success rates with trab-MMC in pseudophakic eyes, probably because of postoperative inflammation after CE.^80^^,^^91^

Tube shunt implantation, an alternative to trabeculectomy, has gained popularity in recent years. The implantation of tube shunts, often referred to as glaucoma drainage devices (GDDs), creates a permanent sclerostomy to drain the aqueous humor into the subconjunctival space.^92^ The advantages of GDDs over trabeculectomy include decreased conjunctival scarring (by diverting aqueous drainage to the equatorial region of the eye and away from the limbus) and the formation of a permanent bleb (plate tube).^92^ Most GDD designs are modeled after the early Molteno implant^93^ and may be valved (promotes unidirectional flow) or nonvalved (passive-acting).^92^ The Ahmed Baerveldt Comparison and Ahmed Versus Baerveldt studies compared the safety and efficacy of the valveless Baerveldt 350-mm^2^ GDD (Johnson & Johnson) to that of the valved Ahmed-FP7 GDD (New World Medical Inc). Both devices were effective in reducing the IOP and the need for IOP-lowering medications, although a favorable IOP decrease, medication burden reduction, and safety profile (but with a higher incidence of hypotony) were seen with the valveless Baerveldt 350-mm^2^ GDD at 5 years.^94^ Recent advancements in valveless GDD operation include the development of the Ahmed ClearPath GDD (New World Medical Inc) and PAUL glaucoma implant (PGI; Advanced Ophthalmic Innovations). The Ahmed ClearPath GDD has several unique design features, such as a flexible, low-lying plate with anterior suture points to increase the ease of implantation, and a prethreaded 4-0 polypropylene ripcord to mitigate the risk of hypotony that has been reported in other GDD studies.^95^ The PGI GDD has a smaller plate that occupies less space in the ACA and a relatively large endplate surface area through which the aqueous humor can be absorbed.^96^ Early outcome data with the Ahmed ClearPath GDD and PGI found mean IOP reductions of 43%^97^ and 51.6%,^96^ at 6 months, respectively.

Both trabeculectomy and GDD implantation are effective treatment options for refractory glaucoma—eyes with poor results after both pharmacotherapy and laser. A 5-year comparison of trabeculectomy and tube shunt operation found that both techniques effectively lower the IOP (trabeculectomy: 49.5%; tube: 41.4%), with the tube group having a better safety profile.^97^ In surgically naïve eyes with refractory glaucoma, the Primary Tube vs Trabeculectomy study found trabeculectomy to be superior,^98^ whereas the Tube vs Trabeculectomy study reported similar outcomes in both groups at 5 years postoperatively in eyes that were not surgically naïve; however, eyes in the tube group had lower failure and reoperation rates.^17^^,^^97^ Frequent complications within the early postoperative period included choroidal effusion (Tube, 14%; Trab, 13%) and shallow anterior chamber (Tube, 10%; Trab, 10%), and late postoperative complications included persistent corneal edema (Tube, 16%; Trab, 9%) and bleb encapsulation (Tube, 2%; Trab, 6%).^17^ Many of the eyes needed postoperative interventions (Tube: 25%, Trab: 70%).^17^ Craven et al^16^ estimated that 25% of patients treated with trabeculectomy or a tube shunt needed additional interventions to address surgical failure.

### Minimally Invasive Glaucoma Surgeries

The potential complications and surgical failures seen with traditional incisional operations speak to the need for better procedures for mild-to-moderate glaucoma that are minimally invasive yet durable. This has led to the introduction of MIGSs, which have revolutionized glaucoma care over the past decade. This group of novel procedures may sufficiently lower the IOP to delay or minimize the need for traditional incisional procedures,^82^ and they are more suitable for patients with mild-to-moderate glaucoma. Minimally invasive glaucoma surgeries can be performed together with cataract operation, which makes them a valuable option for glaucomatous eyes with advanced cataracts (from aging, phacomorphic glaucoma, traumatic glaucoma, etc). Unlike the traditional filtration procedures, MIGSs are relatively simple to perform because they require surgical skills similar to those required for modern-day cataract surgery,^99^ and they can be performed by cataract surgeons who are not glaucoma fellowship trained. Minimally invasive glaucoma surgeries have favorable safety profiles and are less invasive than traditional incisional operations.^100^

One of the management challenges with performing MIGSs lies in whether to bypass or enhance the conventional aqueous outflow^101^ because the currently available MIGS devices target 1 of the 3 pressure-lowering mechanisms: (1) the trabecular outflow pathway, referring to “angle-based” MIGSs that reroute the aqueous flow toward the Schlemm canal; (2) the subconjunctival space, referring to MIGSs that create a drainage pathway, diverting the aqueous humor to the subconjunctival space; (3) the suprachoroidal space, referring to MIGSs that increase the uveoscleral pathway outflow and divert the aqueous flow toward the suprachoroidal space.^100^

### MIGSs Targeting the Trabecular Outflow Pathway

Approximately 50%-75% of the outflow resistance lies within the TM and the inner wall of the Schlemm canal, whereas the remainder resides within the Schlemm canal and its distal collector channels.^102^, ^103^, ^104^, ^105^ This identifies the conventional outflow pathway as an attractive first target for the treatment of glaucoma. Angle-based MIGSs take advantage of the lower resistance within the Schlemm canal and divert the aqueous flow to the canal, thereby bypassing most of the outflow resistance. Despite this, however, a significant proportion of outflow resistance remains, thereby making these procedures unsuitable for patients with severe glaucoma who require significant IOP reduction.^80^ Minimally invasive glaucoma surgeries that target the trabecular outflow pathway fall within the categories of trabecular bypass implant, ab-interno canaloplasty, ab-interno trabeculotomy (AIT), goniotomy, and the more recently introduced combined goniotomy/viscodilation and trabeculotomy/viscodilation procedures.

The iStent (Glaukos Corporation), the first trabecular bypass implant, has produced excellent results when implanted into glaucomatous eyes that are well-controlled on 1 IOP-lowering medication.^80^ Additional IOP lowering is observed when placing more than 1 stent, which led to the development of the iStent inject and iStent inject W.^100^ A study comparing the early outcomes of the iStent and iStent inject reported favorable IOP (iStent, 4.3%; iStent inject, 19.1%) and medication reduction results (iStent, 72.2%; iStent inject, 94.1%) in the iStent inject group at 12 months, with a similar safety profile observed in both the groups.^106^ Ab-interno canaloplasty is typically performed with the iTrack microcatheter (Nova Eye Medical), and a retrospective comparison with ab-externo canaloplasty (iTrack with a 9-0 prolene tensioning suture) found comparable safety and efficacy.^107^ Ab-interno trabeculotomy and goniotomy procedures bring the anterior chamber, Schlemm canal, and distal collector channels into direct communication through the disruption or partial excision of the TM.^108^ The Trabectome (Neomedix), a long-standing AIT procedure, uses electrocauterization to ablate the TM and has been documented to safely and effectively reduce the IOP.^108^ Recent advancements in excisional goniotomy include the Kahook Dual Blade (KDB; New World Medical) and KDB Glide (New World Medical) devices. Although limited data on KDB Glide exist within the literature, several studies of KDB have shown that it has a favorable safety profile and similar effectiveness to AIT procedures.^109^^,^^110^

Angle-based MIGS procedures are easy to perform and have favorable safety profiles, but compared with traditional trabeculectomy, they have more limited abilities to lower IOP.^101^^,^^111^ Distal outflow (collector channels and episcleral veins), which is often overlooked in the treatment of glaucoma, may play a pivotal role in IOP control and is unaffected by canalicular-based MIGS procedures. Studies with bovine and monkey eyes have found that collector channels may alter the pressure distribution within the Schlemm canal, suggesting that the aqueous outflow may depend on the location of these distal elements.^84^^,^^102^^,^^112^ Resistance within the Schlemm canal and the collector channels appears to limit the outflow increase of trabecular bypass procedures to 13%-26% and IOP reduction to the mid-teens, but a greater pressure decrease is expected if a moderate dilation of the Schlemm canal and the collector channels is achieved.^84^^,^^113^^,^^114^ Goniotomy and trabeculotomy may be performed concurrently with the implantation of an ophthalmic viscosurgical device (STREAMLINE Surgical Systems, New World Medical; OMNI360 Surgical Systems, Sight Sciences) to the Schlemm canal to reduce the distal outflow resistance and promote further IOP reduction. Interim analyses of the STREAMLINE and OMNI trials have shown effective, sustained IOP reductions and meaningful medication reductions at 6 and 12 months, respectively.^115^^,^^116^

### MIGSs Targeting the Subconjunctival Space

Minimally invasive glaucoma surgeries devices within this category work similarly to trabeculectomy by diverting the aqueous humor flow directly into the subconjunctival space.^100^ The main disadvantage of this strategy is the potential for subconjunctival fibrosis, which for trabeculectomy may be prevented by the intraoperative application of MMC.^100^ Subconjunctival MIGS devices, which are designed based on the Hagen-Poiseuille equation, include the ab-internally implanted XEN45 gel stent (Allergan) and the ab-externally implanted PRESERFLO microshunt (Santen). Both devices produce comparable safety profiles, IOP reductions, and overall surgical success at 2 years.^117^ The analysis of both implantation approaches with an experimental microfluidic system found higher outflow resistance and less predictable bleb formation with ab-interno implantation. This may affect the long-term IOP control and could direct the development of future subconjunctival-based MIGS devices.^118^

### MIGSs Targeting the Suprachoroidal Space

The third category of MIGSs aims to increase the uveoscleral outflow.^100^ The uveoscleral pathway is not limited by the pressure “floor” formed by episcleral venous pressure; thus, diverting the aqueous humor into the suprachoroidal space could have a greater lower IOP capacity.^119^ Unfortunately, current studies have yet to realize such results. After the recall of CyPass (Alcon) in 2018 because of corneal endothelial cell loss from malpositioned devices, most suprachoroidal MIGSs are still under investigation.^119^ A review of recent studies indicates favorable safety profiles and effective short-term IOP reductions to the mid-teens with the iStent SUPRA (Glaukos Corporation), STARflo (iSTAR Medical), and gold implant (SOLX, Inc). Longer follow-ups and more robust trial designs are still required for the US Food and Drug Administration approval of suprachoroidal MIGSs,^120^ and long-term efficacy may be limited by fibroblast migration and proliferation.^121^

## Conclusion

The pathogenesis of glaucoma is multifactorial and incompletely understood, and diagnosis methods and management strategies are constantly being improved. Treatment outcomes, safety profiles, and recovery times have improved with the introduction of MIGSs. Future work should aim to develop MIGS devices with greater IOP-lowering capabilities than traditional incisional operations.

## Potential Competing Interests

The authors report no competing interests.

## References

[1] Kang J.M., Tanna A.P. (2021). Glaucoma. Med Clin North Am.

[2] Tham Y.C., Li X., Wong T.Y., Quigley H.A., Aung T., Cheng C.Y. (2014). Global prevalence of glaucoma and projections of glaucoma burden through 2040: a systematic review and meta-analysis. Ophthalmology.

[3] Weinreb R.N., Aung T., Medeiros F.A. (2014). The pathophysiology and treatment of glaucoma: a review. JAMA.

[4] Hollands H., Johnson D., Hollands S., Simel D.L., Jinapriya D., Sharma S. (2013). Do findings on routine examination identify patients at risk for primary open-angle glaucoma? The rational clinical examination systematic review. JAMA.

[5] Stein J.D., Khawaja A.P., Weizer J.S. (2021). Glaucoma in adults-screening, diagnosis, and management: a review. JAMA.

[6] Sunderland D.K., Sapra A. (2022). StatPearls [Internet].

[7] Sit A.J., Liu J.H. (2009). Pathophysiology of glaucoma and continuous measurements of intraocular pressure. Mol Cell Biomech.

[8] Goel M., Picciani R.G., Lee R.K., Bhattacharya S.K. (2010). Aqueous humor dynamics: a review. Open Ophthalmol J.

[9] Khazaeni B., Khazaeni L. (2022). StatPearls [Internet].

[10] Stowell C., Burgoyne C.F., Tamm E.R., Ethier C.R. (2017). Lasker/IRRF Initiative on Astrocytes and Glaucomatous Neurodegeneration Participants. Biomechanical aspects of axonal damage in glaucoma: a brief review. Exp Eye Res.

[11] Flammer J., Orgül S., Costa V.P. (2002). The impact of ocular blood flow in glaucoma. Prog Retin Eye Res.

[12] Helmer C., Malet F., Rougier M.B. (2013). Is there a link between open-angle glaucoma and dementia? The Three-City-Alienor cohort. Ann Neurol.

[13] Ko F., Muthy Z.A., Gallacher J. (2018). Association of retinal nerve fiber layer thinning with current and future cognitive decline: a study using optical coherence tomography. JAMA Neurol.

[14] Rodgers C.D., Meyer A.M., Rosenberg N.C. (2018). The impact of conjunctival flap method and drainage cannula diameter on bleb survival in the rabbit model. PLoS One.

[15] Lee D.A., Higginbotham E.J. (2005). Glaucoma and its treatment: a review. Am J Health Syst Pharm.

[16] Craven E.R., Singh I.P., Yu T.M., Rhoten S., Sadruddin O.R., Sheybani A. (2022). Reoperation rates and disease costs for primary open-angle glaucoma patients in the United States treated with incisional glaucoma surgery. Ophthalmol Glaucoma.

[17] Gedde S.J., Herndon L.W., Brandt J.D. (2012). Postoperative complications in the Tube Versus Trabeculectomy (TVT) study during five years of follow-up. Am J Ophthalmol.

[18] Brandão L.M., Grieshaber M.C. (2013). Update on minimally invasive glaucoma surgery (MIGS) and new implants. J Ophthalmol.

[19] Kwon Y.H., Fingert J.H., Kuehn M.H., Alward W.L. (2009). Primary open-angle glaucoma. N Engl J Med.

[20] Vajaranant T.S., Wu S., Torres M., Varma R. (2012). The changing face of primary open-angle glaucoma in the United States: demographic and geographic changes from 2011 to 2050. Am J Ophthalmol.

[21] Stamer W.D., Braakman S.T., Zhou E.H. (2015). Biomechanics of Schlemm's canal endothelium and intraocular pressure reduction. Prog Retin Eye Res.

[22] Simcoe M.J., Weisschuh N., Wissinger B., Hysi P.G., Hammond C.J. (2020). Genetic heritability of pigmentary glaucoma and associations with other eye phenotypes. JAMA Ophthalmol.

[23] Farrar S.M., Shields M.B. (1993). Current concepts in pigmentary glaucoma. Surv Ophthalmol.

[24] Elhawy E., Kamthan G., Dong C.Q., Danias J. (2012). Pseudoexfoliation syndrome, a systemic disorder with ocular manifestations. Hum Genomics.

[25] Łukasik U., Kosior-Jarecka E., Wróbel-Dudzińska D., Kustra A., Milanowski P., Żarnowski T. (2020). Clinical features of pseudoexfoliative glaucoma in treated polish patients. Clin Ophthalmol.

[26] Kalogeropoulos D., Sung V.C. (2018). Pathogenesis of uveitic glaucoma. J Curr Glaucoma Pract.

[27] Sung V.C.T., Barton K. (2004). Management of inflammatory glaucomas. Curr Opin Ophthalmol.

[28] Schlote T., Rohrbach M. (2005). Traumatische Glaukome – eine Ubersicht. Klin Monbl Augenheilkd.

[29] Feroze K.B., Khazaeni L. (2022). StatPearls [Internet].

[30] Fabre-Guillevin E., Tchen N., Anibali-Charpiat M.F., Calluaud L., Ravaud A. (1999). Taxane-induced glaucoma. Lancet.

[31] Pradhan Z.S., Kuruvilla A., Jacob P. (2015). Surgical management of glaucoma secondary to idiopathic elevated episcleral venous pressure. Oman J Ophthalmol.

[32] Wright C., Tawfik M.A., Waisbourd M., Katz L.J. (2016). Primary angle-closure glaucoma: an update. Acta Ophthalmol.

[33] Stefan C., Iliescu D.A., Batras M., Timaru C.M., De Simone A. (2015). Plateau iris–diagnosis and treatment. Rom J Ophthalmol.

[34] Dave S.D., Meyer J.J. (2022). StatPearls [Internet].

[35] Zhang N., Wang J., Chen B., Li Y., Jiang B. (2020). Prevalence of primary angle closure glaucoma in the last 20 years: a meta-analysis and systematic review. Front Med (Lausanne).

[36] Rodrigues G.B., Abe R.Y., Zangalli C. (2016). Neovascular glaucoma: a review. Int J Retina Vitreous.

[37] Jain I.S., Gupta A., Dogra M.R., Gangwar D.N., Dhir S.P. (1983). Phacomorphic glaucoma—management and visual prognosis. Indian J Ophthalmol.

[38] Denis P. (2007). Le glaucome du syndrome irido-cornéo-endothélial. J Fr Ophtalmol.

[39] Masoomian B., Saatchi M., Ghassemi F., Riazi-Esfahani H., Vahedian Z. (2020). Angle closure glaucoma secondary to enlarged Soemmering ring that is clinically similar to iris tumour. Int Med Case Rep J.

[40] Ah-Kee E.Y., Egong E., Shafi A., Lim L.T., Yim J.L. (2015). A review of drug-induced acute angle closure glaucoma for non-ophthalmologists. Qatar Med J.

[41] Cutolo C.A., Bonzano C., Scotto R. (2021). Moving beyond the slit-lamp gonioscopy: challenges and future opportunities. Diagnostics (Basel).

[42] Feng R., Luk S.M.H., Wu C.H.K., Crawley L., Murdoch I. (2019). Perceptions of training in gonioscopy. Eye (Lond).

[43] Porporato N., Baskaran M., Husain R., Aung T. (2020). Recent advances in anterior chamber angle imaging. Eye (Lond).

[44] Leite M.T., Sakata L.M., Medeiros F.A. (2011). Managing glaucoma in developing countries. Arq Bras Oftalmol.

[45] Leske M.C., Connell A.M.S., Wu S.Y. (2001). Incidence of open-angle glaucoma: the Barbados Eye Studies. The Barbados Eye Studies Group. Arch Ophthalmol.

[46] Brusini P., Salvetat M.L., Zeppieri M. (2021). How to measure intraocular pressure: an updated review of various tonometers. J Clin Med.

[47] Nakamura M., Darhad U., Tatsumi Y. (2006). Agreement of rebound tonometer in measuring intraocular pressure with three types of applanation tonometers. Am J Ophthalmol.

[48] Jose J., Ve R.S., Pai H.V. (2020). Agreement and repeatability of Icare ic100 tonometer. Indian J Ophthalmol.

[49] Gao F., Liu X., Zhao Q., Pan Y. (2017). Comparison of the iCare rebound tonometer and the Goldmann applanation tonometer. Exp Ther Med.

[50] Machiele R., Motlagh M., Patel B.C. (2022). StatPearls [Internet].

[51] Cohen L.P., Pasquale L.R. (2014). Clinical characteristics and current treatment of glaucoma. Cold Spring Harb Perspect Med.

[52] Weinreb R.N., Khaw P.T. (2004). Primary open-angle glaucoma. Lancet.

[53] Schuster A.K., Erb C., Hoffmann E.M., Dietlein T., Pfeiffer N. (2020). The diagnosis and treatment of glaucoma. Dtsch Arztebl Int.

[54] Hood D.C., Tsamis E., Bommakanti N.K. (2019). Structure-function agreement is better than commonly thought in eyes with early glaucoma. Invest Ophthalmol Vis Sci.

[55] ICD-10-CM quick reference guide for glaucoma. American Academy of Ophthalmology. https://www.aao.org/Assets/a51b5857-fb6b-4187-a477-48a9095452ee/637358783050770000/glaucoma-icd-10-quick-reference-100120-final-pdf. Accessed June 29, 2022.

[56] Gedde S.J., Vinod K., Wright M.M. (2021). Primary open-angle glaucoma preferred practice Pattern. Ophthalmology.

[57] Ostler E., Rhee D., Burney E., Sozeri Y. (2021). Advances in medical therapy for glaucoma. Curr Opin Ophthalmol.

[58] Lindén C., Alm A. (1999). Prostaglandin analogues in the treatment of glaucoma. Drugs Aging.

[59] Johnson M., McLaren J.W., Overby D.R. (2017). Unconventional aqueous humor outflow: a review. Exp Eye Res.

[60] Cavet M.E., Vittitow J.L., Impagnatiello F., Ongini E., Bastia E. (2014). Nitric oxide (NO): an emerging target for the treatment of glaucoma. Invest Ophthalmol Vis Sci.

[61] Weinreb R.N., Scassellati Sforzolini B.S., Vittitow J., Liebmann J. (2016). Latanoprostene bunod 0.024% versus timolol maleate 0.5% in subjects with open-angle glaucoma or ocular hypertension: the Apollo Study. Ophthalmology.

[62] Medeiros F.A., Martin K.R., Peace J., Scassellati Sforzolini B., Vittitow J.L., Weinreb R.N. (2016). Comparison of latanoprostene bunod 0.024% and timolol maleate 0.5% in open-angle glaucoma or ocular hypertension: the LUNAR study. Am J Ophthalmol.

[63] Faiq M.A., Wollstein G., Schuman J.S., Chan K.C. (2019). Cholinergic nervous system and glaucoma: from basic science to clinical applications. Prog Retin Eye Res.

[64] Aslam S., Gupta V. (2022). StatPearls [Internet].

[65] Brooks A.M., Gillies W.E. (1992). Ocular beta-blockers in glaucoma management. Clinical pharmacological aspects. Drugs Aging.

[66] Zimmerman T.J. (1993). Topical ophthalmic beta blockers: a comparative review. J Ocul Pharmacol.

[67] Denis P., Lafuma A., Berdeaux G. (2008). Costs and persistence of alpha-2 adrenergic agonists versus carbonic anhydrase inhibitors, both associated with prostaglandin analogues, for glaucoma as recorded by the United Kingdom General Practitioner Research Database. Clin Ophthalmol.

[68] Tanna A.P., Johnson M. (2018). Rho kinase inhibitors as a novel treatment for glaucoma and ocular hypertension. Ophthalmology.

[69] Kahook M.Y., Serle J.B., Mah F.S. (2019). Long-term safety and ocular hypotensive efficacy evaluation of netarsudil ophthalmic solution: rho kinase elevated IOP treatment trial (ROCKET-2). Am J Ophthalmol.

[70] Serle J.B., Katz L.J., McLaurin E. (2018). Two phase 3 clinical trials comparing the safety and efficacy of netarsudil to timolol in patients with elevated intraocular pressure: rho kinase elevated IOP treatment trial 1 and 2 (ROCKET-1 and ROCKET-2). Am J Ophthalmol.

[71] Schwartz G.F., Quigley H.A. (2008). Adherence and persistence with glaucoma therapy. Surv Ophthalmol.

[72] Samples J.R., Singh K., Lin S.C. (2011). Laser trabeculoplasty for open-angle glaucoma: a report by the American Academy of Ophthalmology. Ophthalmology.

[73] Freitas A.L., Ushida M., Almeida I. (2016). Selective laser trabeculoplasty as an initial treatment option for open-angle glaucoma. Arq Bras Oftalmol.

[74] Tsang S., Cheng J., Lee J.W. (2016). Developments in laser trabeculoplasty. Br J Ophthalmol.

[75] Babighian S., Caretti L., Tavolato M., Cian R., Galan A. (2010). Excimer laser trabeculotomy *vs* 180 degrees selective laser trabeculoplasty in primary open-angle glaucoma. A 2-year randomized, controlled trial. Eye (Lond).

[76] Lee J.R., Choi J.Y., Kim Y.D., Choi J. (2011). Laser peripheral iridotomy with iridoplasty in primary angle closure suspect: anterior chamber analysis by pentacam. Korean J Ophthalmol.

[77] Ritch R., Tham C.C., Lam D.S. (2007). Argon laser peripheral iridoplasty (ALPI): an update. Surv Ophthalmol.

[78] Fu J., Qing G.P., Wang N.L., Wang H.Z. (2013). Efficacy of laser peripheral iridoplasty and iridotomy on medically refractory patients with acute primary angle closure: a three year outcome. Chin Med J (Engl).

[79] Anand N., Klug E., Nirappel A., Solá-Del Valle D. (2020). A review of cyclodestructive procedures for the treatment of glaucoma. Semin Ophthalmol.

[80] Kalarn S., Le T., Rhee D.J. (2022). The role of trabeculectomy in the era of minimally invasive glaucoma surgery. Curr Opin Ophthalmol.

[81] Vinod K., Gedde S.J., Feuer W.J. (2017). Practice preferences for glaucoma surgery: a survey of the American Glaucoma Society. J Glaucoma.

[82] Overby D.R., Stamer W.D., Johnson M. (2009). The changing paradigm of outflow resistance generation: towards synergistic models of the JCT and inner wall endothelium. Exp Eye Res.

[83] Mäepea O., Bill A. (1992). Pressures in the juxtacanalicular tissue and Schlemm’s canal in monkeys. Exp Eye Res.

[84] Swaminathan S.S., Oh D.J., Kang M.H., Rhee D.J. (2014). Aqueous outflow: segmental and distal flow. J Cataract Refract Surg.

[85] Binibrahim I.H., Bergström A.K. (2017). The role of trabeculectomy in enhancing glaucoma patient’s quality of life. Oman J Ophthalmol.

[86] Rotchford A.P., King A.J. (2010). Moving the goal posts definitions of success after glaucoma surgery and their effect on reported outcome. Ophthalmology.

[87] Al Habash A., Aljasim L.A., Owaidhah O., Edward D.P. (2015). A review of the efficacy of Mitomycin C in glaucoma filtration surgery. Clin Ophthalmol.

[88] Kansal V., Armstrong J.J., Hutnik C.M. (2020). Trends in glaucoma filtration procedures: a retrospective administrative health records analysis over a 13-year period in Canada. Clin Ophthalmol.

[89] Pantalon A., Feraru C., Tarcoveanu F., Chiselita D. (2021). Success of primary trabeculectomy in advanced open angle glaucoma. Clin Ophthalmol.

[90] Mathew R.G., Parvizi S., Murdoch I.E. (2019). Success of trabeculectomy surgery in relation to cataract surgery: 5-year outcomes. Br J Ophthalmol.

[91] Ogata-Iwao M., Inatani M., Takihara Y., Inoue T., Iwao K., Tanihara H. (2013). A prospective comparison between trabeculectomy with Mitomycin C and phacotrabeculectomy with Mitomycin C. Acta Ophthalmol.

[92] Wang J., Barton K. (2017). Aqueous shunt implantation in glaucoma. Taiwan J Ophthalmol.

[93] Melamed S., Fiore P.M. (1990). Molteno implant surgery in refractory glaucoma. Surv Ophthalmol.

[94] Christakis P.G., Zhang D., Budenz D.L. (2017). Five-year pooled data analysis of the Ahmed Baerveldt comparison study and the Ahmed versus Baerveldt study. Am J Ophthalmol.

[95] Grover D.S., Kahook M.Y., Seibold L.K. (2022). Clinical outcomes of Ahmed ClearPath implantation in glaucomatous eyes: a novel valveless glaucoma drainage device. J Glaucoma.

[96] Vallabh N.A., Mason F., Yu J.T.S. (2022). Surgical technique, perioperative management and early outcome data of the PAUL® glaucoma drainage device. Eye (Lond).

[97] Gedde S.J., Schiffman J.C., Feuer W.J. (2012). Treatment outcomes in the Tube Versus Trabeculectomy (TVT) study after five years of follow-up. Am J Ophthalmol.

[98] Gedde S.J., Feuer W.J., Lim K.S. (2020). Treatment outcomes in the primary Tube versus trabeculectomy study after 3 years of follow-up. Ophthalmology.

[99] Fellman R.L., Mattox C., Singh K. (2020). American Glaucoma Society position paper: microinvasive glaucoma surgery. Ophthalmol Glaucoma.

[100] Pereira I.C.F., van de Wijdeven R., Wyss H.M., Beckers H.J.M., den Toonder J.M.J. (2021). Conventional glaucoma implants and the new MIGS devices: a comprehensive review of current options and future directions. Eye (Lond).

[101] Lusthaus J.A., Meyer P.A.R., Khatib T.Z., Martin K.R. (2020). The effects of trabecular bypass surgery on conventional aqueous outflow, visualized by hemoglobin video imaging. J Glaucoma.

[102] Battista S.A., Lu Z., Hofmann S., Freddo T., Overby D.R., Gong H. (2008). Reduction of the available area for aqueous humor outflow and increase in meshwork herniations into collector channels following acute IOP elevation in bovine eyes. Invest Ophthalmol Vis Sci.

[103] Johnson M. (2006). What controls aqueous humour outflow resistance?. Exp Eye Res.

[104] Rosenquist R., Epstein D., Melamed S., Johnson M., Grant W.M. (1989). Outflow resistance of enucleated human eyes at two different perfusion pressures and different extents of trabeculotomy. Curr Eye Res.

[105] Grant W.M. (1963). Experimental aqueous perfusion in enucleated human eyes. Arch Ophthalmol.

[106] Guedes R.A.P., Gravina D.M., Lake J.C., Guedes V.M.P., Chaoubah A. (2019). One-year comparative evaluation of iStent or iStent inject implantation combined with cataract surgery in a single center. Adv Ther.

[107] Gallardo M.J., Supnet R.A., Ahmed I.I.K. (2018). Circumferential viscodilation of Schlemm’s canal for open-angle glaucoma: ab-interno vs ab-externo canaloplasty with tensioning suture. Clin Ophthalmol.

[108] Schehlein E.M., Kaleem M.A., Swamy R., Saeedi O.J. (2017). Microinvasive glaucoma surgery: an evidence-based assessment. Expert Rev Ophthalmol.

[109] Barry M., Alahmadi M.W., Alahmadi M., AlMuzaini A., AlMohammadi M. (2020). The safety of the Kahook Dual Blade in the surgical treatment of glaucoma. Cureus.

[110] Dorairaj S., Tam M.D., Balasubramani G.K. (2019). Twelve-month outcomes of excisional goniotomy using the Kahook Dual Blade® in eyes with angle-closure glaucoma. Clin Ophthalmol.

[111] Durr G.M., Töteberg-Harms M., Lewis R., Fea A., Marolo P., Ahmed I.I.K. (2020). Current review of Excimer laser Trabeculostomy. Eye Vis (Lond).

[112] Lu Z., Zhang Y., Freddo T.F., Gong H. (2011). Similar hydrodynamic and morphological changes in the aqueous humor outflow pathway after washout and Y27632 treatment in monkey eyes. Exp Eye Res.

[113] Zhou J., Smedley G.T. (2005). A trabecular bypass flow hypothesis. J Glaucoma.

[114] Zhou J., Smedley G.T. (2006). Trabecular bypass: effect of schlemm canal and collector channel dilation. J Glaucoma.

[115] Hirsch L., Cotliar J., Vold S. (2021). Canaloplasty and trabeculotomy ab interno with the OMNI system combined with cataract surgery in open-angle glaucoma: 12-month outcomes from the ROMEO study. J Cataract Refract Surg.

[116] Lazcano-Gomez G., Garg S.J., Yeu E., Kahook M.Y. (2022). Interim analysis of STREAMLINE® surgical system clinical outcomes in eyes with glaucoma. Clin Ophthalmol.

[117] Scheres L.M.J., Kujovic-Aleksov S., Ramdas W.D. (2021). XEN® Gel Stent compared to PRESERFLO™ MicroShunt implantation for primary open-angle glaucoma: two-year results. Acta Ophthalmol.

[118] Lee R.M.H., Bouremel Y., Eames I., Brocchini S., Khaw P.T. (2019). The implications of an ab interno versus ab externo surgical approach on outflow resistance of a subconjunctival drainage device for intraocular pressure control. Transl Vis Sci Technol.

[119] Shah M. (2019). Micro-invasive glaucoma surgery – an interventional glaucoma revolution. Eye Vis (Lond).

[120] Kammer J.A., Mundy K.M. (2015). Suprachoroidal devices in glaucoma surgery. Middle East Afr J Ophthalmol.

[121] Vinod K. (2018). Suprachoroidal shunts. Curr Opin Ophthalmol.

